# A comprehensive study on genome-wide coexpression network of KHDRBS1/Sam68 reveals its cancer and patient-specific association

**DOI:** 10.1038/s41598-019-47558-x

**Published:** 2019-07-31

**Authors:** B. Sumithra, Urmila Saxena, Asim Bikas Das

**Affiliations:** Department of Biotechnology, National Institute of Technology Warangal, Warangal, 506004 Telangana India

**Keywords:** Cancer genomics, Gene regulatory networks

## Abstract

Human KHDRBS1/Sam68 is an oncogenic splicing factor involved in signal transduction and pre-mRNA splicing. We explored the molecular mechanism of KHDRBS1 to be a prognostic marker in four different cancers. Within specific cancer, including kidney renal papillary cell carcinoma (KIRP), lung adenocarcinoma (LUAD), acute myeloid leukemia (LAML), and ovarian cancer (OV), KHDRBS1 expression is heterogeneous and patient specific. In KIRP and LUAD, higher expression of KHDRBS1 affects the patient survival, but not in LAML and OV. Genome-wide coexpression analysis reveals genes and transcripts which are coexpressed with KHDRBS1 in KIRP and LUAD, form the functional modules which are majorly involved in cancer-specific events. However, in case of LAML and OV, such modules are absent. Irrespective of the higher expression of KHDRBS1, the significant divergence of its biological roles and prognostic value is due to its cancer-specific interaction partners and correlation networks. We conclude that rewiring of KHDRBS1 interactions in cancer is directly associated with patient prognosis.

## Introduction

Human KHDRBS1 (KH domain-containing, RNA-binding, signal transduction-associated protein 1) gene encodes Sam68 (Src substrate associated in mitosis 68 kDa), a member of STAR (signal transduction activator of RNA) family of RNA-binding proteins^1,2^. Sam68 is mainly involved for pre-mRNA splicing and signal transduction pathway in cells. It is required in mRNA export and stability as well as it participates in apoptosis, mitosis, and cell cycle progression^3^. The function of Sam68 is highly regulated by cell signaling pathway, thus provides the link between signaling and mRNA splicing. The dual function of Sam68 is due to the presence of highly conserved KH-domain and Src homology domain (SH-domain, specifically SH2 and SH3 domain), which are involved in RNA binding and signal transduction pathway respectively^1,4^. Therefore external cues could influence the splicing pattern of the Sam68 target gene. Matter *et al*.^5^ have shown that phosphorylation of Sam68 via ERK pathway modulates the alternative splicing of CD44 gene. Evidently in a cancer cell, RNA splicing machinery receives aberrant signaling response via Sam68 and results in the generation of oncogenic splicing variant^5–8^. Higher expression of Sam68/KHDRBS1 is shown to play significant role in various cancer cells, such as, colon^9^, prostate^10^, renal^11^, colorectal^12^, breast^13^, esophageal squamous cell carcinoma^6^ neuroblastoma^14^ bladder cancer^15^ renal cell carcinoma^11^, cervical cancer^7^ hepatic cancer^16^ and non-small lung cancer cells^17^. It is also identified as a prognostic marker in a few cancer tissues^11,15^. However, we argue that higher expression of KHDRBS1/Sam68 may not be a reason for cancer phenotype in all types of tissues because cancer arises due to the perturbation of multiple genes. Moreover, none of the previous findings have shown the molecular basis of KHDRBS1/Sam68 to be a prognostic marker. Based on existing observation, we ask whether higher expression of KHDRBS1 always affect the patient survival and is there any evidence at the level of the molecular network, which expressly supports KHDRBS1 as the prognostic marker. To counter our queries, we selected four human cancer of different tissues, which are kidney renal papillary cell carcinoma (KIRP), lung adenocarcinoma (LUAD), acute myeloid leukemia (LAML), and ovarian cancer (OV). We used high throughput gene and transcript level data from the cancer genome atlas (TCGA) for this study. Our analysis shows that expression of KHDRBS1 within a specific cancer is heterogeneous and higher expression of KHDRBS1 does not always affects the patient survival in all cancer. To understand the differential behavior, we have done the genome-wide correlation analysis to find coexpressed genes and transcripts with KHDRBS1. Our results show that the coexpressed genes and transcripts form the functional clusters which are majorly involved in cancer progression in LUAD and KIRP but not in LAML and OV. Our finding suggests that the clinical outcomes of higher expression of KHDRBS1 depend on context-specific molecular interaction network which could be an essential parameter to design personalized medicine.

## Results

### Heterogeneous expression of KHDRBS1 mRNA in the cancer patient

**Figure 1:**
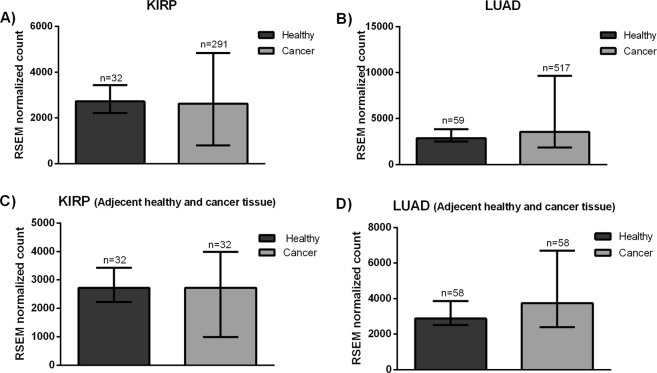
Expression of KHDRBS1 mRNA in KIRP and LUAD: (**A**,**B**) mRNA expression in the healthy and cancerous tissue of KIRP & LUAD patients. (**C**,**D**) mRNA expression in adjacent healthy and cancer tissue from a same patient in KIRP & LUAD respectively (Error bar in each diagram represent the maximum and minimum value of RSEM normalized count. KIRP: kidney renal papillary cell carcinoma, LUAD: lung adenocarcinoma).

To understand expression status of KHDRBS1, we have compared the KHDRBS1 mRNA expression level in healthy and cancerous tissue of KIRP and LUAD patients. We obtained TCGA RNA sequencing data from BROAD Institute (http://gdac.broadinstitute.org/). RNA-Sequencing by Expectation-Maximization (RSEM) values of KHDRBS1 expression was taken for comparison. We found that expression of KHDRBS1 is highly scattered in cancer tissue in both KIRP and LUAD (Fig. 1A,B). To reconfirm our observation, we have compared the KHDRBS1 expression in healthy and cancer tissue of the same patient. Similarly, we observed there is no observable difference of KHDRBS1 expression in cancer compared to normal (Fig. 1C,D). The healthy adjacent tissue sample for LAML and OV is not available in TCGA. Therefore to compare the KHDRBS1expression in healthy and cancer patients, we collected data from GEO (Gene expression omnibus) and explored KHDRBS1expression level in OV (GSE18520)^18^ and LAML (GSE9476)^19^ [Supplementary Fig. S1A,B]. Here, we observed there is no difference of KHDRBS1expression level, which is similar to KIRP and LUAD (Fig. 1A,B). However, theses GEO datasets are not used for further analysis in this article. Based on this observation we decided to group the cancer patients depending on KHDRBS1 expression level (higher and lower expression). Higher and lower expression is classified based on the Z-score value of KHDRBS1 expression, which is provided by TCGA for all four cancer types i.e. KIRP, LUAD, OV and LAML.

**Figure 2:**
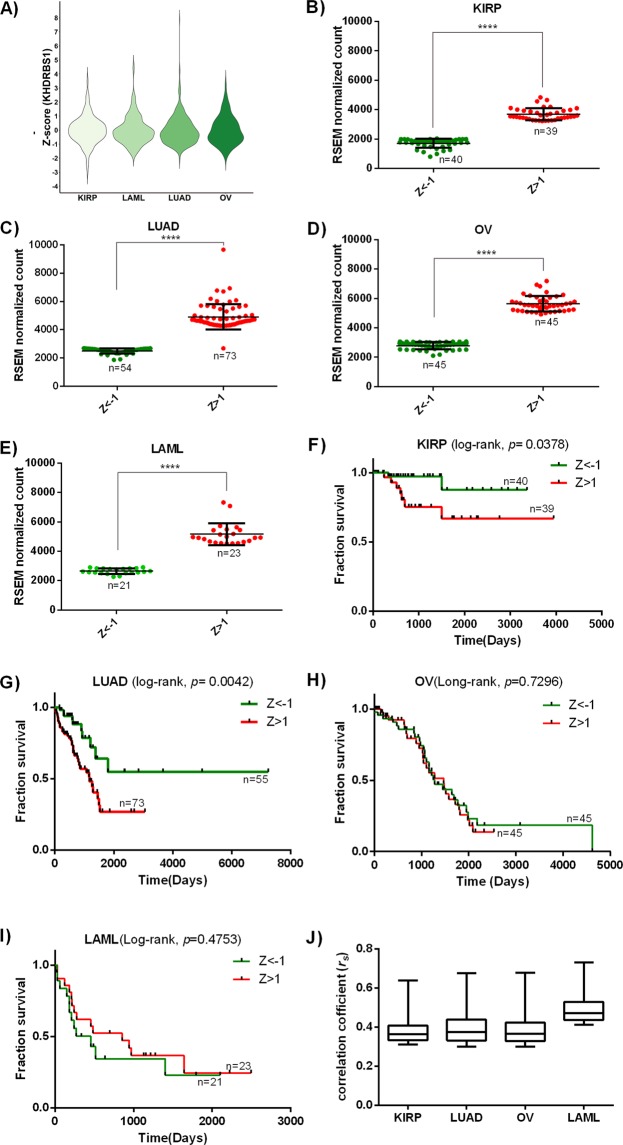
Patient specific expression of KHDRBS1, survival and correlation analysis: (**A**) Volcano plot summarizing the Z-score distribution of KHDRBS1expression in different cancer. (**B**–**E**) shows the difference in KHDRBS1 mRNA expression level in Z > 1 and Z < −1 sample in KIRP, LUAD, OV and LAML respectively (****P < 0.0001). (**F**–**I**) Kaplan-Meier curve shows the comparison of fraction survival in higher expression (Z > 1) and lower expression (Z < −1) group in all four cancer. In KIRP and LUAD, the higher expression of KHDRBS1 affects the patient survival (P < 0.05), whereas in OV and LAML there is no difference in patient survival (P > 0.05) in higher and lower expression group. (**J**) Boxplot summarizing the distribution of correlation coefficient of KHDRBS1 to all other genes (*r*_*s*_ > 0.3, P < 0.05). In boxplot, the median is indicated by the horizontal line dividing the interquartile range (Q25, Q75). Upper and lower ticks represent the maximum and minimum value (KIRP: kidney renal papillary cell carcinoma, LUAD: lung adenocarcinoma, LAML: acute myeloid leukemia, and OV: ovarian cancer).

It is observed that in all four cancers the Z -score of KHDRBS1 expression is widely distributed from negative to positive values (Fig. 2A). This indicates that the expression of KHDRBS1 mRNA is not recurrently high or low in all cancers. Furthermore, Z-score distribution also shows that there are many patients within specific cancer who have significantly high or low expression of KHDRBS1. This suggests that KHDRBS1 expression is patient-specific and not cancer-specific. Therefore higher and lower expression of KHDRBS1 within a particular cancer type is grouped based on Z -score of greater than 1 (higher expression) or less than −1 (low expression) respectively (Supplementary Fig. S2A). Simultaneously we observed that Z-score of KHDRBS1 expression is not widely distributed in normal adjacent tissue compared to the cancerous tissue of KIRP and LUAD (Supplementary Fig. S2B). Therefore the RSEM values of KHDRBS1 mRNA Z > 1 and Z < −1 are screened for cancer tissue of four type of cancer, and non-parametric Mann-Whitney test was performed to check whether patients within Z > 1 and Z < −1 group have any significant difference in KHDRBS1 mRNA expression level. Figure 2B–E shows in KIRP, LUAD, LAML and OV, there is statistically significant (P < 0.0001) difference in expression among the patients with Z > 1 and Z < −1. However, this stratification of patients in higher and lower expression based on Z-score of KHDRBS1 expression is limited to specific cancer patients within a particular cancer type.

### Higher expression of KHDRBS1 correlates with patient survival in KIRP and LUAD

To understand the clinical outcomes of KHDRBS1 higher expression in cancer patients, we performed survival analysis using Kaplan-Meier survival curve and log-rank test^20^. Patient-specific clinical data was collected from TCGA clinical data set, and survival was compared between two group i,e Z > 1 and Z < −1 of KHDRBS1. Survival analysis shows higher expression of KHDRBS1 (Z > 1) significantly reduces (P < 0.05) the patient survival in KIRP, and LUAD (Fig. 2F,G). However, in LAML and OV, higher expression of KHDRBS1 does not show any difference (P > 0.05) in patient survival rate (Fig. 2H,I). This result shows that higher expression of KHDRBS1 has the prognostic value in KIRP and LUAD for a specific group of cancer patients, but not in LAML and OV. Further, in LAML and OV, the expression of KHDRBS1 is significantly (P < 0.0001) high in the patients with Z > 1 as compared to Z < −1, although the higher expression does not affect the patient survival. This gives us fascinating evidence that the over-expression of KHDRBS1 may not always be accountable for cancer progression and patient survival. The cellular function of a gene or protein depends on its interacting partners. In this scenario, the interacting partners of KHDRBS1 in LUAD and KIRP are possibly different from LAML and OV, which results in a different outcome. Moreover, each cancer has a unique phenotypic property which is evolved due to distinct molecular interaction inside a cell. Therefore, investigation on the interacting partners of KHDRBS1 and correlation among them could light-up exact mechanism of KHDRBS1 function in cancer.

### Genome-wide coexpression analysis and functional clustering of KHDRBS1 coexpressed genes

**Figure 3:**
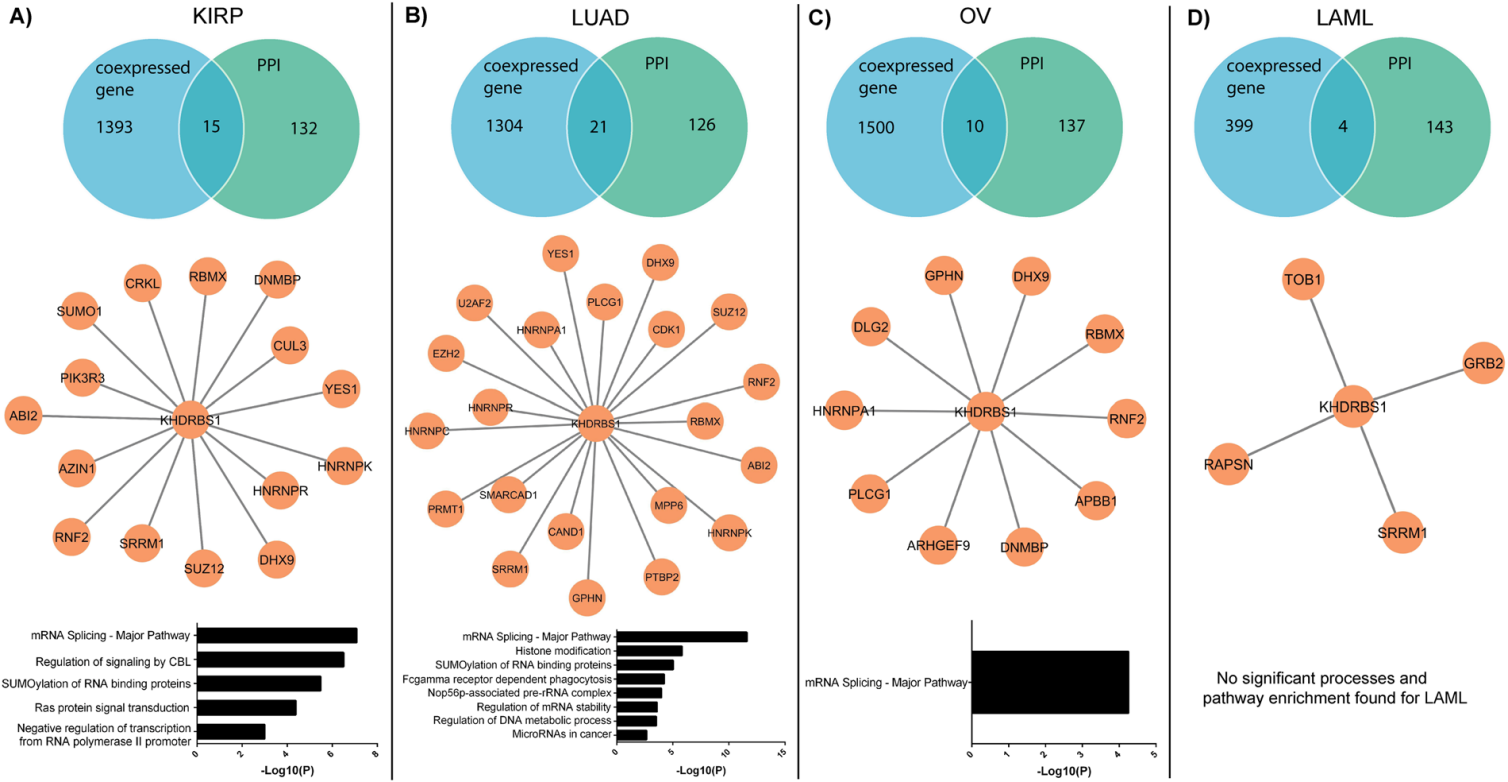
Overlap of protein-protein interactions (PPI) dataset and coexpressed gene of Sam68/KHDRBS1 and processes and pathway enrichment analysis in different cancer: (**A**–**D**) Venn diagram and network figure shows the overlapping genes which coexpress and interact with Sam68/KHDRBS1in KIRP, LUAD, OV and LAML respectively. The bar diagram indicates the process and pathway enrichment analysis of overlapping gene in respective cancer. Logarithmic corrected p-values for significant overrepresentation are shown.

**Figure 4:**
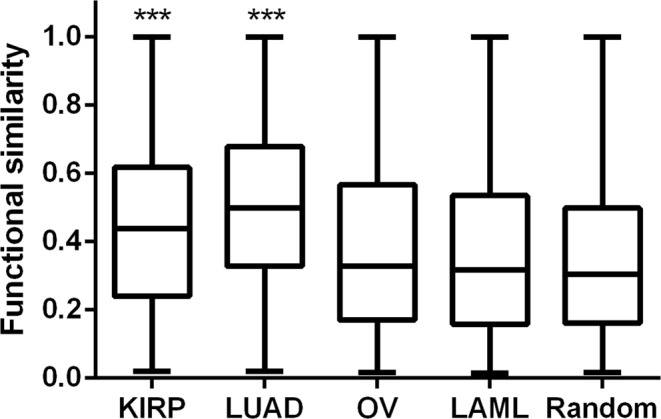
Distribution of functional similarities between the coexpressed genes in different cancer. The functional similarities between coexpressed genes (*r*_*s*_ > 0.3, p < 0.05) with KHDRBS1 is calculated based on GO semantic similarity. The random set of genes (Random) is used as negative control. The functional similarity is high in case of KIRP and LUAD compared to the OV, LAML and random set (n = 500) of genes (box boundaries represent the first and third quartile (Q.25, Q.75). The median is indicated by the horizontal line dividing the interquartile range. Upper and lower ticks represent the maximum and minimum value). Mann-Whitney test was performed separately in between KIRP vs. OV, LAML, Random and LUAD vs. OV, LAML, Random (***P < 0.001).

To address the patient and cancer-specific role of KHDRBS1, we performed genome-wide correlation analysis. We calculated the correlation of KHDRBS1 to all other genes (20531 genes) expressed in specific cancer. For each type of cancer, patients with higher KHDRBS1 expression (Z > 1) were selected for correlation analysis. Genes with correlation coefficient (*r*_*s*_) > 0.3 and P < 0.05 were selected for further analysis. Distribution of correlation coefficient (*r*_*s*_ > 0.3 and P < 0.05) (Fig. 2J) shows the median values for KIRP, LUAD and OV are almost equal, but it is high in case of LAML. However, the higher number of correlated genes in LAML does not play any significant role in the overall function, because in the subsequent experiment (Fig. 4) we have observed that the functional similarity between these genes is less. Next, we constructed protein interaction map of KHDRBS1/Sam68, and we selected direct physical interactions between other human protein and KHDRBS1/Sam68 from databases^21–26^. We considered experimentally determined binary interactions, which are generated using yeast two-hybrid or high-throughput experiments (Supplementary Table S1). Genes with the correlation coefficient (*r*_*s*_) > 0.3, P < 0.05 and which have physical interaction with KHDRBS1 were screened for each cancer. Both criteria were chosen to increase the stringency of selection of KHDRBS1 interacting partners in a specific cancer cell. Venn diagrams (Fig. 3) show that each cancer type has overlapping genes which are coexpressed and also physically interact with KHDRBS1. Network in Fig. 3 shows, most of these coexpressed and interacting genes of KHDRBS1 are different across the four cancers. Moreover, we observed that numbers of these overlapping genes are less in OV and LAML compared to KIRP and LUAD. However, to understand the cancer-specific biological function of these genes, the process and pathway enrichment analysis were performed. We observed that in case of KIRP and LUAD the cancer-specific processes such as regulation of signaling by cbl^27^ SUMOylation of RNA binding protein^28–30^, ras protein signal transduction pathway^31^, microRNAs in cancer^32^ are predominant (Fig. 3). However, in case of OV we only observed that pathway of RNA splicing is an only predominant event and no process or pathway enrichment is found in case of LAML. It is interesting to notice that overexpression of KHDRBS1 leads to enrichment of cancer-specific events in KIRP, LUAD but not in OV and LAML. The result indicates a positive correlation between KHDRBS1 expression status and cancer phenotype in KIRP and LUAD. The results also show a similar expression pattern of a gene differentially affects the disease state, probably due to cancer and patient-specific genetic profile. Therefore genes which are coexpressed and interact with KHDRBS1 are mostly different in KIRP and LUAD, although they are involved in cancer-specific biological processes which are accountable for patient mortality.

A common observation in gene expression is that many genes which show similar expression patterns frequently clustered according to their biological functions^33,34^. Therefore analysis of functional clustering of all genes which are co-expressed with KHDRBS1 can provide a clear view of predominant functions associated with the group of genes expressed in a specific cellular context. Next, we have done protein-protein interaction enrichment analysis for all coexpressed genes (*r*_*s*_ > 0.3, P < 0.05) in each cancer using Metascape tools, which fetch the interaction data from BioGrid^23^, InWeb_IM^35^, and OmniPath^36^. The resulting network was again used to identify densely connected network components using molecular complex detection (MCODE) algorithm^37^. Pathway and process enrichment analysis find the function of each densely connected component (Supplementary Fig. S3). The result shows that coexpressed genes in KIRP and LUAD are mostly involved in cell cycle, and cell division related processes such as chromatin assembly and organization, cell cycle checkpoint control. As many of these densely connected genes are co-expressed with KHDRBS1, it can be presumed that probably KHDRBS1 is also involved in a similar function in KIRP and LUAD. However, in OV and LAML, the network components are less densely connected and several gene clusters which are present in KIRP and LUAD and involved in cell proliferation are absent in OV and LAML (Supplementary Fig. S3). It is now comprehensible that KHDRBS1 driven molecular processes are similar in case of KIRP and LUAD but different in OV and LAML for a specific group of patients. We then examined whether the genes which are coexpressed with KHDRBS1 are involved in similar biological functions or not. Gene Ontology (GO) semantic similarity was used to quantify the functional association of coexpressed genes. We found that coexpressed genes in KIRP and LUAD tend to have significantly high (P < 0.001) functional relationships compared to OV, LAML and random set (Fig. 4). It explains coexpressed genes in KIRP and LUAD are involved in the functionally similar biological processes and pathways, which support our previous observation of functional clustering of coexpressed genes (Supplementary Fig. S3) as most of the enriched processes in KIRP and LUAD are linked to cell proliferation.

### Genome-wide transcript correlation analysis reconfirms that KHDRBS1/Sam68 is a prognostic marker in KIRP and LUAD

**Figure 5:**
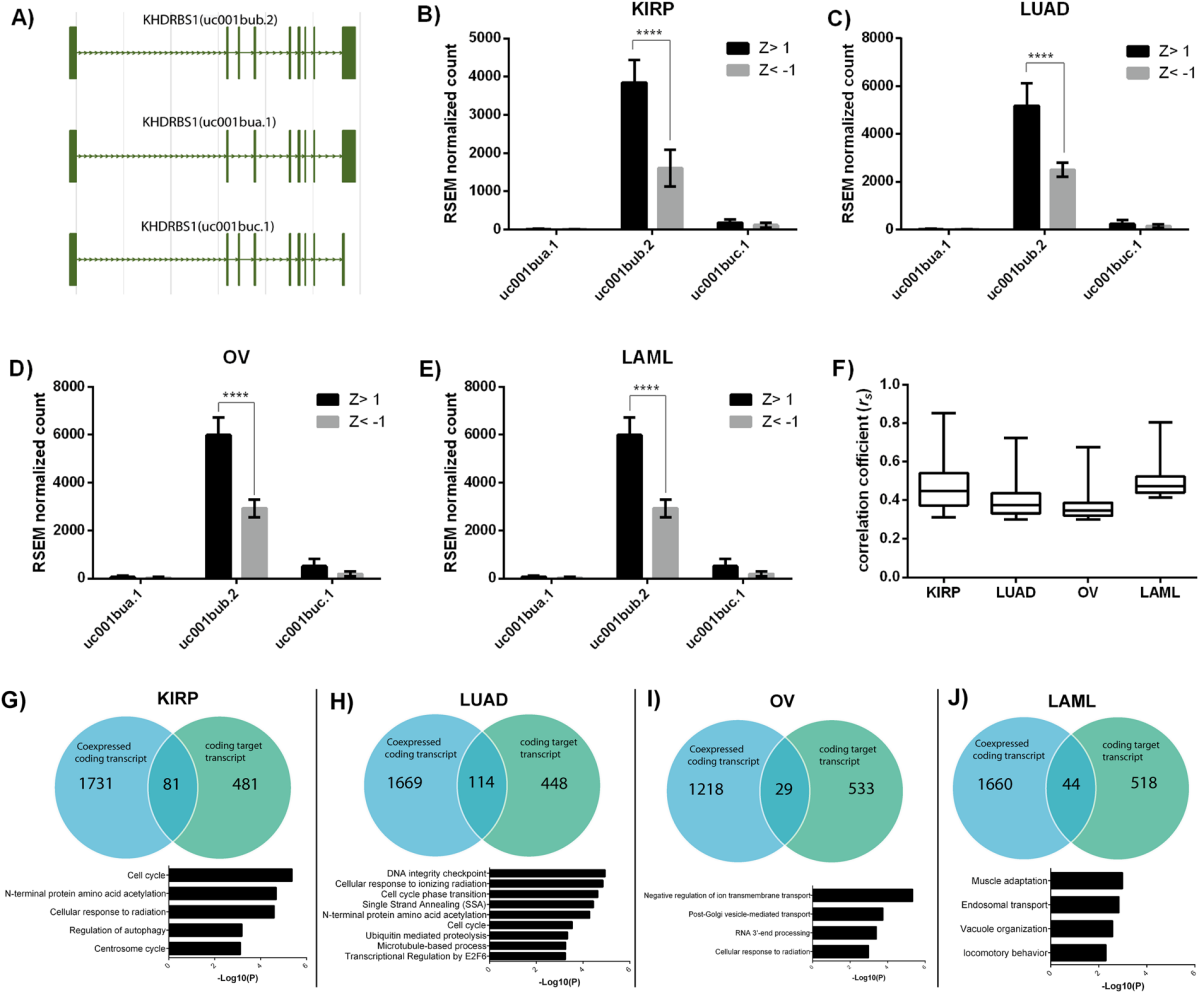
Relative expression of different KHDRBS1 transcript and process and pathway enrichment analysis of coexpressed target transcript of KHDRBS1/Sam68: (**A**) Transcript (uc001bua, uc001bub and uc001buc) structure of KHDRBS1 from UCSC database. (**B**–**D**,**F**) show the relative expression of uc001bua, uc001bub, and uc001buc transcript in KIRP, LUAD, OV, and LAML respectively (error bar represent the standard deviation). (**F**) Boxplot is summarizing the distribution of correlation coefficient of uc001bub with all other transcripts (*r*_*s*_ > 0.3, P < 0.05) in all four cancers. (**G**–**J**) Venn diagram representing overlapping coexpressed and target transcript of KHDRBS1/Sam68. The bar diagram indicates the process and pathway enrichment analysis of overlapping genes in specific cancer (Logarithmic corrected P-values for significant overrepresentation are shown).

In the previous section, we analyzed the gene level expression data, which illustrate the coexpressed genes and their prevailing cellular function in different cancer. However, Sam68 is known as RNA binding protein and involved in RNA splicing. Indeed, Sam68 driven oncogenic isoform is reported in many cancer^5,8^. Therefore investigating the co-regulated target transcript of Sam68 could provide the clues of differential behavior in different cancer cells. Hence we have analyzed the transcript level expression data to identify the co-expressed isoform with KHDRBS1/Sam68. Prior to correlation analysis, we have checked how many different isoform variants present for KHDRBS1. UCSC data shows (Fig. 5A) that KHDRBS1 can be spliced in three different splice isoforms uc001bua, uc001bub, and uc001buc. Next, we examined the relative expression of these isoforms in different cancer datasets. Our result shows, out of three isoforms, uc001bub has higher mean expression level than other isoforms in all cancer. Additionally, uc001bub expression is significantly high in Z > 1 compared to Z < −1 samples in all cancer (Fig. 5B–E). This suggests that higher expression of KHDRBS1 is mainly contributed by uc001bub isoform. Based on this result we calculated the Spearman correlation coefficient (*r*_*s*_) between uc001bub and all transcripts (73,599 transcripts). We examined the pattern of association of uc001bub transcript to all other transcripts in all four cancers, but there was no observable trend (Fig. 5F). Next, top 2000 transcripts with correlation coefficient (*r*_*s*_) > 0.3 and P < 0.05 were screened for each cancer type. However, many of these UCSC transcripts do not code for protein. Therefore to identify the protein-coding transcript, we have matched the UCSC transcript to RefSeq accession number of NCBI, and subsequently, coding transcripts were chosen for analysis.

To find the target transcripts which are co-expressed with uc001bub, the genome-wide binding region of Sam68 was obtained from RNA complete experiment by Ray *et al*.^38^. The study shows that Sam68 can bind to total 268 sites in the human genome (human genome version hg19). From the co-ordinate of the binding region and using hg19 as the reference genome, we predicted that total 1036 different transcripts could be produced by Sam68 (Supplementary Fig. S4). We also found that out of 1036 transcripts, 562 are coding transcripts. Target transcripts (coding), which are present in top 2000 correlated transcript data were screened and subjected to process and pathway enrichment analysis (Fig. 5G–J). We notice similar result like gene-level data, coexpressed target transcript of Sam68 are involved in cancer-specific processes such as cell cycle, protein N-terminal acetylation, cell cycle phase transition, E2F6 transcription regulation in KIRP and LUAD^39–41^. However, in OV and LAML, the cancer linked biological processes are absent (Fig. 5I,J, bar diagram).

Next, we examined all highly correlated transcripts (*r*_*s*_ > 0.6, P < 0.05) for process and pathway enrichment analysis using Metascape tools. We observed that coexpressed transcripts in KIRP and LUAD are mostly involved in cell division, and proliferation, which are highly interconnected (Supplementary Fig. S5A,B). However, in LAML (Supplementary Fig. S5C), prevailing pathway and processes are not directly linked to the cancer-specific events, and in OV we did not find any process enrichment. The results of both gene and transcript level correlation analysis show that even though the KHDRBS1 expression pattern is same in KIRP, LUAD, OV, and LAML for specific group of patients, its higher expression has different clinical outcomes due to the change in interaction partners and correlation network. Our study shows molecular network of KHDRBS1 is patient-specific and varies across the cancer tissue. The essentiality of a gene in disease progression is determined by its interaction partners^42^. Similarly, our study shows that higher expression & clinical outcomes is not always a proportionally linked event, rather it depends on network architecture in a cell.

## Discussion

In this study, we present genome-scale evidence for KHDRBS1/Sam68 to be a prognostic or non-prognostic marker in four different human cancers. Our result represents that higher expression of a gene is not always a cause of pathogenesis of cancer. A gene can be labelled as prognostic maker if it is involved in crucial molecular processes, which are specific to the disease progression. In the present work, we evaluated the expression level of KHDRBS1 in KIRP, LUAD, LAML and OV cancer. For the first time, we have shown that expression of KHDRBS1 in all four cancers is heterogeneous and patient specific. However, our results show that higher expression of KHDRBS1 causes reduced survival of the patient in KIRP and LUAD but not in LAML and OV. This indicates; in KIRP and LUAD, higher expression of KHDRBS1 possibly plays a critical role in the cancer-specific event. To understand the cancer-specific behavior of KHDRBS1, we performed the genome-wide correlation analysis in all four cancers for the patients with higher expression of KHDRBS1 and screened the genes which have significant correlation and direct interaction with KHDRBS1. It is noticed that the common genes, which are coexpressed and interact with KHDRBS1 are involved in the cancer-specific processes in KIRP and LUAD, but not in LAML and OV. This provides us the lead to do the further experiment to find the cancer-specific module in all coexpressed genes of KHDRBS1. We identified that several recurrent network modules are involved in cell cycle and division linked processes in KIRP and LUAD. These network modules contain a core set of genes, which, when highly expressed are sufficient for cell proliferation and metastasis. Additionally, the functional similarity shows that more significant numbers of coexpressed genes are involved in similar molecular functions in KIRP and LUAD compared to OV and LAML. For an additional layer of understanding, we have calculated the genome-wide correlation of isoform level data as KHDRBS1/Sam68 is involved in RNA splicing. These results also confirm that cancer driven biological processes are enriched in KIRP and LUAD not in LAML and OV, although KHDRBS1 predominant isoform uc001bub is highly expressed in all four cancers. The change of cellular environment drives the rewiring of molecular network of a particular gene which can result in alteration of gene function^43^. We observed a similar result in case of KHDRBS1 in the different cancer cell. It should be noted that the observation is restricted to specific group of patients, either in LUAD or KIRP. This is not generalized observation for specific cancer type rather it is patient-specific. Therefore the present work supports the need of personalized medicine and diagnosis in cancer treatment. In general, a gene is identified as prognostic cancer biomarker when its mRNA expression level is significantly correlated with overall patient survival^44^. Moreover, our observations suggest that besides higher expression; a prognostic biomarker should directly or indirectly be associated with the cancer-specific network and event. Therefore to understand the prognostic value of a target molecule a detailed landscape of possible molecular events should be studied, which will lead to improved cancer diagnosis and therapy.

## Methods

### Datasets and data classifications

The Cancer Genome Atlas (TCGA) RNA sequencing data of KIRP, LUAD, LAML, and OV, with clinical annotations, were retrieved from Broad GDAC Firehose Stddata (http://gdac.broadinstitute.org/). We used level 3 whole transcriptome expression data from ‘illuminahiseq_rnaseqv2-RSEM_isoform_normalized’. For transcript expression, we used normalized “scaled_estimates” RSEM counts of isoforms. The raw data were mapped to the hg19 reference genome assembly^45^. Sample sequencing methods and detailed description of processing can be found from the previous publication^46,47^. We classifies patient samples into two groups based on expression of KHDRBS1 as Z = +1 and above (higher expression of KHDRBS1) and Z = −1 and below (lower expression of KHDRBS1). For example, a sample is said to have high expression of a gene if its expression is at least one standard deviation above its mean expression in the subtype.

### Measurement of coexpression

We computed the Spearman’s rank correlation coefficient to measure the coexpression levels between two genes. It is a nonparametric measure of association. It assesses the nonlinear monotonic relationship between the two variables by the linear relationship between the ranks of the values of the two variables. The following formula is used to find the correlation

$${r}_{s}=\frac{6{\sum }_{i=1}^{n}{d}_{i}^{2}}{n({n}^{2}-1)}$$

where; *d*_*i*_ = the difference between the ranks of the ith observations of the two variables. n = the number of pairs of values. Under the null hypothesis of statistical independence of the variables, for a sufficiently large sample, the quantity

$$t=\frac{{r}_{s}}{\sqrt{(1-{r}_{s}^{2})/(n-2)}}$$

follows a student’s t-distribution with n-2 degree of freedom^48^. We used Hmisc Package in R to calculate the *r*_*s*_ and significance level (P-value).

### Survival analysis

To perform the survival analysis, we collected the clinical data from Broad GDAC Firehose Stddata (http://gdac.broadinstitute.org/) and classified the patients into two groups based on mRNA expression level of KHDRBS1 as Z =  + 1 and above (high) and Z = −1 and below (low). We compared the high and low expression of KHDRBS1 on patient survival using Kaplan and Meier method^49^ and tested for significance using Log-Rank tests. Survival curves were generated using GraphPad Prism 7 software.

### Pathway and process enrichment analysis and transcript annotation

Pathway and process enrichment analysis was carried out using the Metascape tool^50^ with the following ontology sources: GO Biological Processes, KEGG Pathway and Reactome Gene Sets. The transcript annotation was done using hg19 as reference genome, which is available in UCSC genome browser database (http://genome.ucsc.edu).

### Functional semantic similarity between genes

The functional similarity between genes was measured by the semantic similarity between sets of GO terms with which they were annotated. We applied the method proposed by Wang *et al*.^51^ to quantify the functional similarity. Considering two genes G1 and G2 annotated by GO term sets GO1 = [go11, go12, …, go1m] and GO2 = [go21, go22, …, go2n] respectively their semantic similarity score of Wang’s method is defined as:

$${\rm{Sim}}({\rm{G}}1,{\rm{G}}2)=\frac{{\sum }_{1\le {\rm{i}}\le {\rm{m}}}{\rm{Sim}}(g{o}_{1i,}G{O}_{2})+{\sum }_{1\le {\rm{j}}\le {\rm{n}}}{\rm{Sim}}(g{o}_{2j,}G{O}_{1})}{m+n}$$

Semantic similarity score of Wang’s method was calculated using GOSemSim package in R^52^.

### Prediction of target transcript

The genomic coordinates of genome-wide binding sites of sam68 were obtained from previously published RNAcompete pull down assay^38^. We have considered only experimentally determined binding sites. All the binding coordinates were then mapped to corresponding transcripts of hg19 using UCSC Genome browser (http://genome.ucsc.edu/cgi-bin/hgGateway). If the binding coordinates of Sam68 present within a transcript coordinate then we selected that transcript as target transcript. Likewise, we have screened all possible UCSC transcripts which have sam68 binding site.

### Statistical method

The difference in expression level was analyzed using non-parametric Mann-Whitney test. GraphPad Prism 7 software was used for statistical analysis.

### Ethics approval

This article does not contain any studies with human participants or animals performed by any of the authors. Therefore, informed consent is not required.

## Supplementary information

supplementary file

## Data Availability

Cancer patient data sets are retrieved from http://gdac.broadinstitute.org. The datasets generated after analysis during the current study are available from the corresponding author on reasonable request.
