# Supplementary material for: A comprehensive study on genome-wide coexpression network of KHDRBS1/Sam68 reveals its cancer and patient-specific association
Source: Sci Rep. 2019 Jul 31;9:11083. doi: 10.1038/s41598-019-47558-x (PMC6668649; doi:10.1038/s41598-019-47558-x)
Supplement: Supplementary file 1 — supplementary file [file 41598_2019_47558_MOESM1_ESM.pdf]

**A comprehensive study on genome-wide coexpression network of KHDRBS1/Sam68 reveals its cancer and patient-specific association.**

B. Sumithra, Urmila Saxena, Asim Bikas Das*

Department of Biotechnology, National Institute of Technology Warangal, Warangal-506004, India.

*Corresponding author:

Dr. Asim Bikas Das
Assistant Professor
Department of Biotechnology,
National Institute of Technology Warangal,
Warangal 506004, Telangana, India
E-mail: bikasasim@gmail.com, asimbikas@nitw.ac.in,
Tel No: +91-8106311048/+91-8332969440

# Supplementary Figure S1

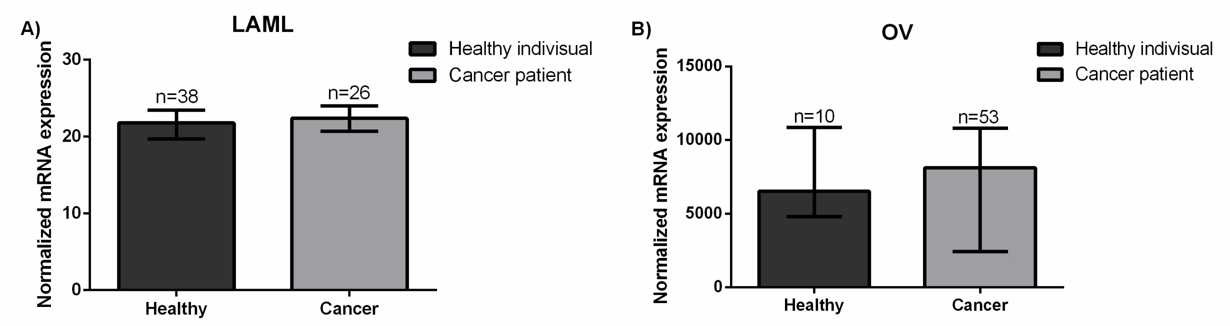


**Supplementary Figure S1:** Expression of KHDRBS1 mRNA in LAML (acute myeloid leukemia) and OV (ovarian carcinoma): A) & B) mRNA expression in the healthy and cancerous tissue of LAML and OV patients respectively. The normalized mRNA expression data of microarray experiment was collected from the Gene Expression Omnibus (Error bar in each diagram represent the maximum and minimum value of normalized mRNA expression).

## Supplementary Figure S2

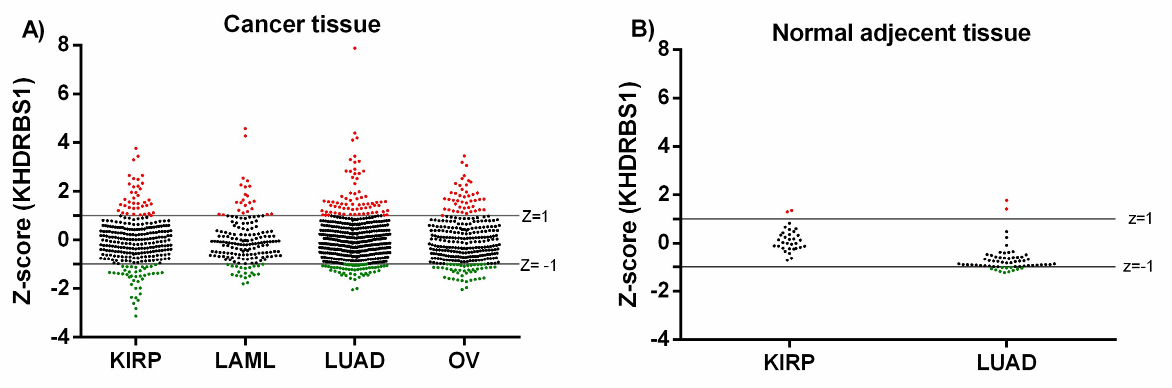


**Supplementary Figure S2**: Z-score distribution of KHDRBS1 expression in cancer and normal adjacent tissue: A) Dot plot summarizing the Z-score distribution of KHDRBS1 expression in four different cancers. The horizontal line indicates the chosen threshold value of Z-score (Z=1 and Z=-1). The red color dots indicates the patient with Z-score>1 and green color indicates the patient with Z-score<-1. B) The dot plot is summarizing the Z-score distribution of KHDRBS1 expression in the normal adjacent tissue of KIRP and LUAD (a similar dot plot for LAML and OV are not shown due to non-availability of normal adjacent tissue data). It is observed that the Z-score distribution of KHDRBS1 expression in the normal adjacent tissue of KIRP and LUAD is not widely distributed compared to cancer tissue.

**Supplementary Table S1**

| |
|---|
| ABI2, ACTB, AGO1, AHI1, AMPH, APBB1, APC, ARHGEF4, ARHGEF9, AZIN1, BAIAP2L1, BMI1, BTK, BZRAP1, CAND1, CASP8, CBL, CBX6, CCDC8, CD2AP, CD2BP2, CD81, CDK1, CIRBP, CREB3L3, CREBBP, CRK, CRKL, CSK, CUL3, CUL7, DDX5, DHX9, DLG1, DLG2, DLG3, DLG4, DNMBP, DOCK2, DOCK3, DROSHA, EFEMP1, EMG1, ESR1, EZH2, FADD, FGR, FNBP4, FRK, FYN, GAS7, GPHN, GRAP, GRAP2, GRB2, HCK, HNRNPA1, HNRNPC, HNRNPK, HNRNPR, INSR, IRS1, ITK, ITSN1, ITSN2, JAK3, LCK, LGR4, LYN, MAPK1, MIA2, MPP6, MYO1C, MYO7A, NCF1, NCK1, NCK2, NCKIPSD, NPHP1, OBSL1, OSTF1, PACSIN1, PACSIN2, PACSIN3, PIK3R1, PIK3R3, PLCG1, POT1, PPP1R13B, PRMT1, PSTPIP1, PTBP2, PTK6, PTPN6, RALY, RAPSN, RASA1, RBFOX2, RBM7, RBMX, RIPK1, RNF2, RPA1, RPA2, RPA3, RUSC2, SASH1, SCG5, SH3GL1, SH3KBP1, SH3PXD2A, SH3YL1, SKAP2, SMAD2, SMARCA2, SMARCAD1, SNRPN, SNX30, SNX9, SORBS1, SPATA13, SRC, SRRM1, SSFA2, STAT3, STUB1, SUMO1, SUZ12, TBL1X, TJP1, TNFRSF1A, TNFSF11, TNS3, TOB1, TUBB3, TUBB4A, U2AF2, UBA52, UBASH3B, UBC, VAV1, VCL, WBP4, YES1, YTHDC1, ZBTB7A, ZDHHC6 |

**Supplementary Table S1:** List of KHDRBS1/Sam68 interacting genes

## Supplementary Figure S3

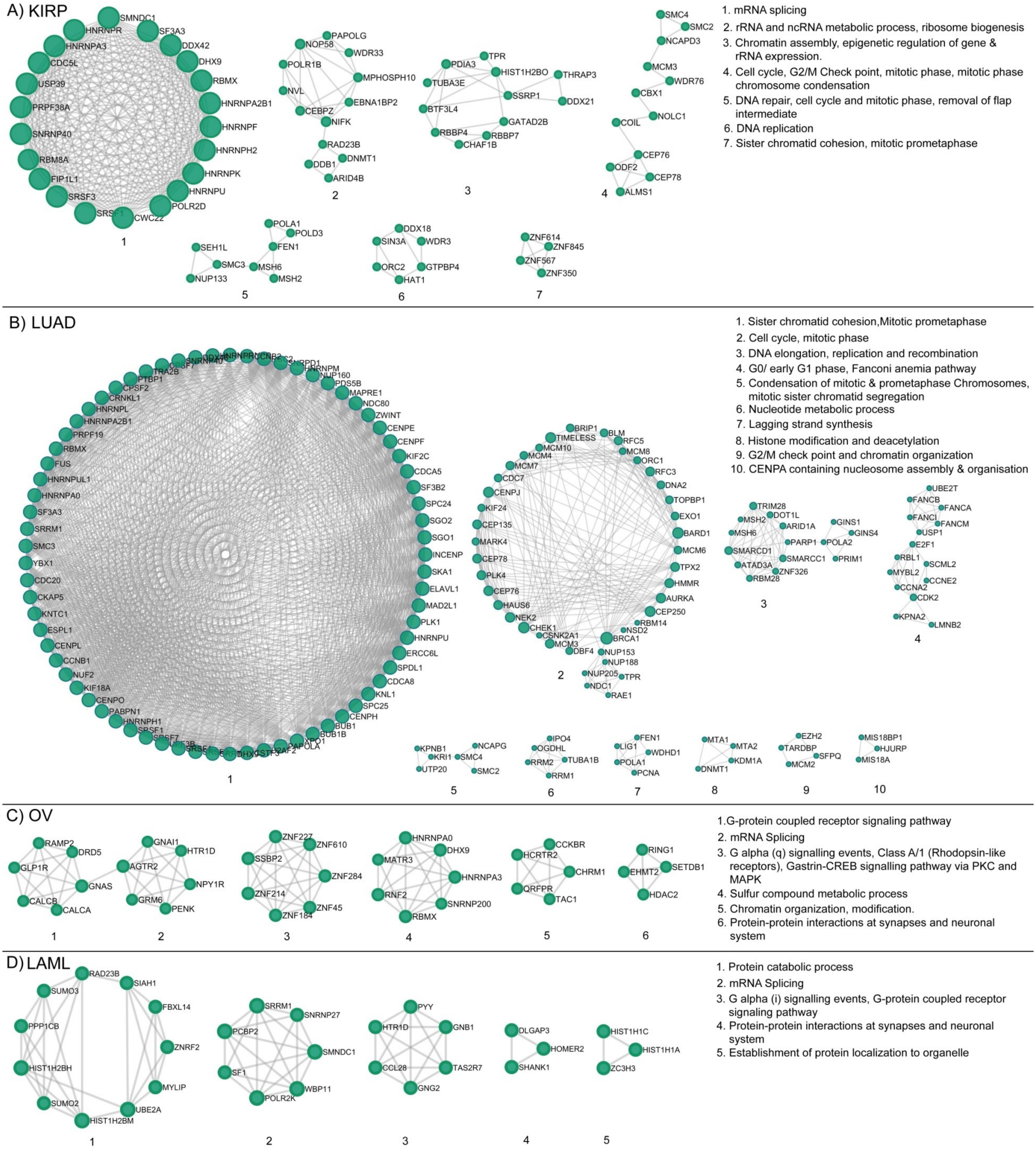


**Supplementary Figure S3** Functional clustering of coexpressed genes in different cancer tissue: A), B), C) and D) show densely connected components in coexpressed gene ($r_s$>0.3, P<0.05) of KHDRBS1/Sam68 in KIRP, LUAD, OV and LAML, and their functions (gene ontology) in each of cancer tissue.

**Supplementary Figure S4**

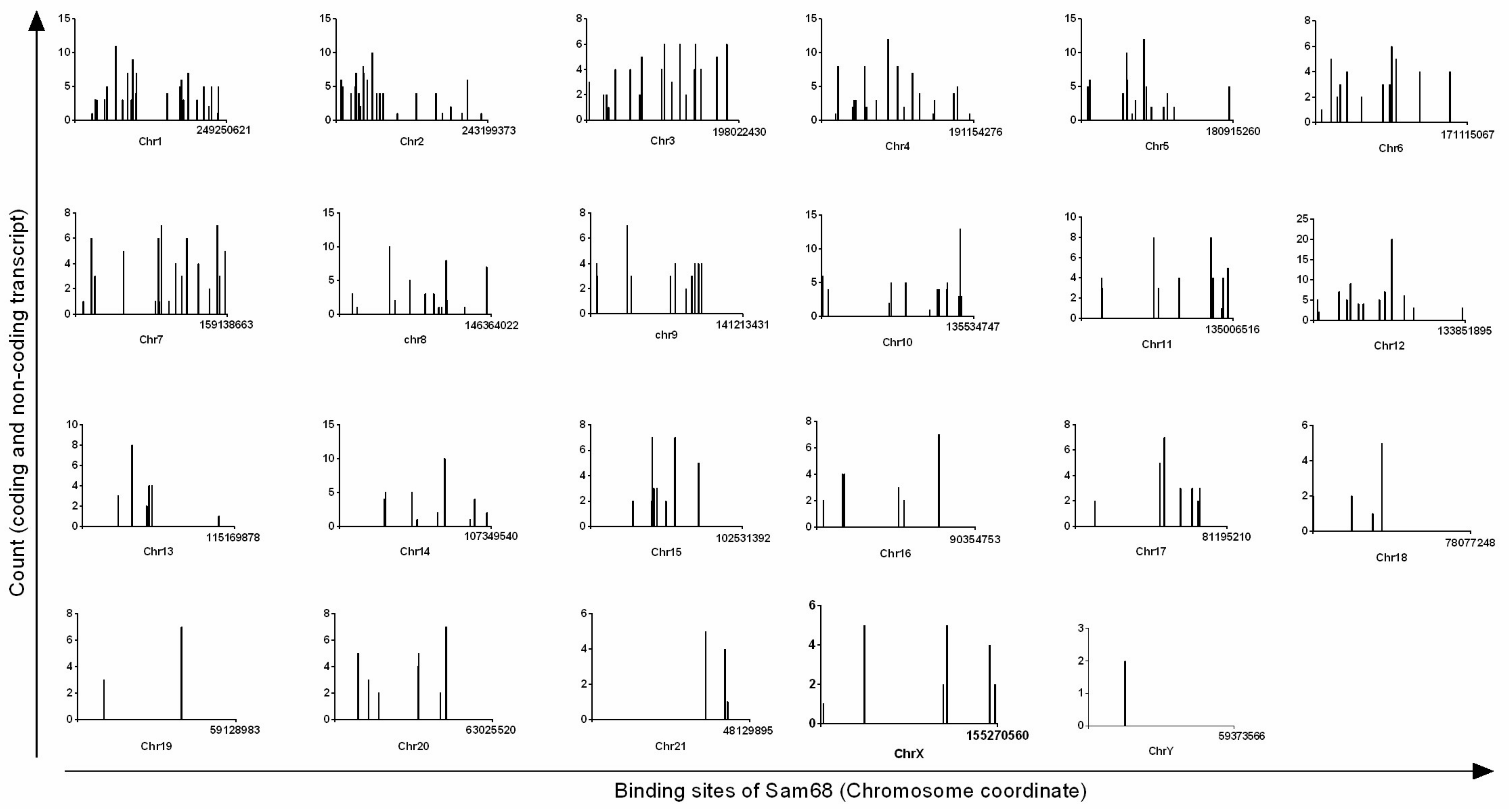


**Supplementary Figure S4** Genome-wide binding region and count of predicted target transcripts of Sam68. Each histogram represents a chromosome and x- axis represents chromosome coordinate. The binding sites of Sam68 are indicated by bar on x-axis and length of the bar corresponds to number of predicted transcripts.

**Supplementary Figure S5**

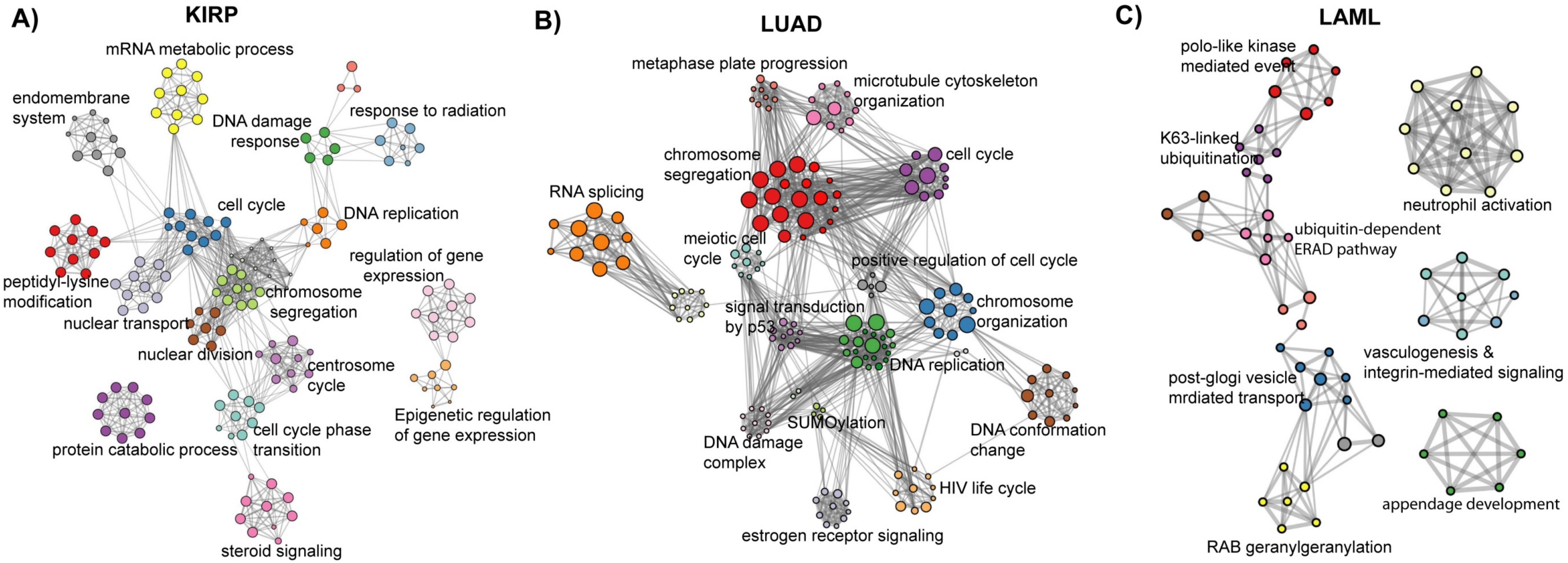


**Supplementary Figure S5** Process and pathway enrichment analysis of highly correlated transcript of KHDRBS1/Sam68**.** A) & B) shows over-represented biological processes, which regulates the cell proliferation such as, cell cycle, chromosome segregation in KIRP and LUAD. C) Over-represented biological processes in LAML are not linked with the cancer-specific event. Due to the insufficient number of highly correlated transcripts ($r_s$>0.6), no process and pathway enrichment is found in OV. The P-value < 0.01, minimum count 3 and enrichment factor > 1.5 are considered for enrichment analysis.
